# Risky Sexual Behavior among Rural Female Adolescents in Malaysia: A Limited Role of Protective Factors

**DOI:** 10.5539/gjhs.v6n3p165

**Published:** 2014-03-23

**Authors:** Maryam Ahmadian, Hanina H. Hamsan, Haslinda Abdullah, Asnarulkhadi Abu Samah, Amna Md Noor

**Affiliations:** 1 Department of Social and Development Sciences, Faculty of Human Ecology, Universiti Putra Malaysia, Selangor, Malaysia; 2 Institute for Social Science Studies, Universiti Putra Malaysia, Selangor, Malaysia

**Keywords:** adolescent, risky sexual behavior, risk and protective factors, Asian

## Abstract

**Purpose:**

This paper presents the findings of a cross-sectional survey on the risk and protective factors of premarital sexual behavior among rural female adolescents in Peninsular Malaysia.

**Methods:**

We investigated data on 770 female respondents aged 13-17 years in rural areas to identify predictive factors for premarital sexual intercourse. Data were analyzed using bivariate and multivariate regression. Specific socio-demographic factors, psychological and family domains, peer delinquency, and knowledge and attitudes about sexuality were considered in risky sexual behaviors in rural Malay girls. The effects of other covariates for premarital sexual intercourse were controlled by logistic regression model.

**Results:**

Of the 770 rural female students, about 3.2% of respondents reported experience of sexual intercourse in the past three months. Out of those sexually active girls, 36% were 17 years old and 20% stated having sexual intercourse with more than one partner, and 72% did not use contraception during the most recent sexual intercourse. Midnight activities, peer-sexual disorder, self-evaluation, and attitude toward sexual health were significant predictors of sexual intercourse in rural girls in Malaysia.

**Conclusion:**

The finding highlights the impact of psychological factors and peer group influences on the challenges of premarital sexual behavior among rural girls and the notion of school-based sexual health education for adolescents. This study triggers other researchers take into account a comprehensive view of protective factors operating in adolescents’ risky sexual behaviors in Asian culture seeing that family domain variables, unexpectedly, exerted no predicting influence on sexually active female teens in rural areas in Malaysia.

## 1. Introduction

Sexual relations outside of marriage, particularly before 15 years of age raise the prevalence of negative health outcomes. Previous research on adolescents’ sexuality has directed towards identifying risk factors such as peer influence, parental involvement, abuse history, and drug use, which are associated to risky sexual behaviors. Nevertheless, culture as a possible risk or protective factor was almost neglected (Gardner & Wilcox, 1993; Lacky & Moberg, 1998). In addition, research pertaining to Asian culture and adolescents’ risky sexual behavior is inadequate. Yet, the finding of previous studies has indicated that race or ethnicity is one of critical factor associated with sexual behaviors (Schuster et al.,1998).

Studies have also shown that risky sexual behaviors among adolescents can lead to sombre health consequences such as sexually transmitted diseases (STDs), teenage pregnancy, and Human Immunodeficiency Virus (HIV) infection. Previous researchers found that unprotected sexual behavior can cause a series of harmful physical, emotional, and social outcomes, especially for girls (Jejeebhoy et al.,2005). Even the proportion of girls having sexual intercourse before marriage was higher in number in contrast to boys (Liu et al., 2006; Shtarkshall et al.,2009).

Involvement in premarital sexual activities in Malaysia verified in a small number among adolescents. However, in the past two decade, sexual activity among Malaysian adolescents has initiated to get higher as a result of significant societal changes. A total of 18,652 pregnancies among teenagers aged between 10-19 years old were reported by the Ministry of Health Malaysia in 2011 (Annual Report, 2011). A review of the literature shows boys are more likely to be sexually experienced than girls in Malaysia (Brown, 1997; Herold et al.,1992). However, the percentage of unmarried adolescents who have had sexual intercourse was increased (Zulkifli et al.,1995).

The risky sexual incidents among the adolescent reported by the National Study on Reproductive Health of Adolescents in the year 1994-95 were only 1% of the respondents (13 cases from 1379 adolescents aged 15-17 years) who had sexual intercourse (National Population and Family Development Board, 1998). From the 1996 National Health and Morbidity Survey, 1.8% of 30 000 school-going respondents admitted that they had had sexual intercourse. Male teens were twice as likely to be sexually involved as female students (2.5% vs. 1.2%). Of those who had sexual intercourse, 19.9% were homosexual and 9.4% had sex with prostitutes (World Health Organization Regional Office for the Western Pacific, 2005). It is difficult to compare prevalence rates of sexual intercourse among adolescents with the findings of previous studies at the local level due to bias against certain groups such as certain ethnicity, gender or ages.

Previous research focusing on risky sexual behavior has found that variables such as gender, employment, and sexual attitudes were associated with sexual intercourse among adolescents. For example, a less conservative attitudes score was linked with five times greater likelihood of being sexually experienced than a conservative score (Zulkifli & Low, 2000). Whereas few national and international research papers exists documenting the adolescents’ sexual practices in Malaysia, there is no research report on adolescents’ risky sexual behavior among rural female students. Thus, the overarching goal of this research is to systematically examine the determinants of risky sexual behaviors among rural female teens without considering ethnicity.

The theoretical framework guiding the research on the development of risk and protective factors was based on Social Developmental Model (SDM) proposed by Catalano and Hawkins (1996). Reviewed literature has shown that this model had been used in several discrete studies (Catalano et al., 1996; Lonczak et al., 2001; Yi et al., 2010; Harliza et al.,2012). In this study, an integrated approach to the assessment of risk and protective factors was viewed as causes of sexual risky behaviour, particularly sexual intercourse among adolescents. The buffer model was already applied in determining the new paradigm of risk/protection which reduces the impact of risk factors (Armstrong et al.,2005). Based on the Social Developmental Model (SDM), sexual attitudes and behaviours among adolescents are learned through all these socialization agents namely, parents, friends, school, and community.

This paper specifically intends to scrutinize risk and protective factors influencing risky sexual behaviours in rural female adolescents at the individual, peer, family, school, community, and social realms. This study employed a survey design to explore predictive factors that concern sexual intercourse among school-attending female adolescents. Socio-psychological theory was used as a guiding ground to adjust behavioral facets of health among individuals and communities (Ahmadian et al.,2012). The results will contribute to health education to reduce sexual risk behaviour among unmarried adolescents in Asian countries. The term of rural teen/rural student/rural girl/female teen/female adolescent in this paper refers to rural female adolescent aged 13-17 years.

## 2. Methodology

The cross-sectional survey was conducted from January to March 2012 at 41 schools located in ten states of Peninsular Malaysia. The study used a multistage cluster random sampling method to acquire 770 rural female adolescents aged 13 to 17 years which were drawn from a total of population of 993,220 students according to the latest 2010 census of Malaysia. A list of all schools was obtained from the Ministry of Education (MOE) in Malaysia. The ratio of students’ number in rural and urban areas was 30:70 percent. 41schools out of 72 schools were selected for data collection. Students within the schools were randomly selected and all of them agreed to join in the study. The Bahasa Malaysia version of the instrument was given to the participants. The response rate was 100% with no refusals. Students completed the online survey by logging on the selected website via internet or ad-hoc server for school with limited internet accessibility. Questionnaires were administered online and students filled out questionnaires at computer labs. The instrument also had the offline versions of the equal test.

The female adolescents were assured of confidentiality and enumerators left the labs during completion of the survey. Approximately 3% of all female adolescents in rural areas were selected reflecting a representative sample of rural female adolescents in Malaysia. Inclusion criteria for subject selection were: (1) students who live in rural areas and communities; (2) rural female adolescents aged 13 to 17 years; (3) capable of completing questionnaires on and off-line.

Approval to perform the survey was provided by the Research Management Centre (RMC) at University of Putra Malaysia. Letters authorizing data collections at the participating schools were obtained prior to the survey. Trained data collectors were executed to collect the data in the selected schools. Data collectors clarified the aim of the study to the adolescents before data collection process.

Statistical Package for Social Science version 19 (IBM, Chicago, IL, USA) software was used to analyze the data. Bivariate regression and multiple logistic regression were done by using the enter method in order to determine the risk and protective factors influencing risky sexual behavior along with premarital sexual intercourse. Multivariate analysis examined several predictors of sexual intercourse among rural female teens, particularly controlled the effects of other covariates by logistic regression model for premarital sexual intercourse.

These analyses estimate whether the beta coefficient of variables influencing early and risky sexual behaviours connects any higher odds for adolescents having premarital sexual intercourse. A two tailed p-value of 0.05 was used to determine the statistical significance for all examinations. Standardized Cronbach’s for the scales and sub-scales in the instrument ranged from .60 to .70. Since none of the independent variables had standard error larger than 2.0, there was no proof of multicollinearity.

### 2.1 Questionnaire

The questionnaire consists of sections which include socio-demographic characteristics, sexually risky behaviors, knowledge and attitudes towards sexuality, psychological domain, family domain, peer delinquency, and sexual activity.

#### 2.1.1 Socio-Demographic Characteristics

The aspect covered age, marital status of parents, head of household, head of household’s education level, and living arrangement.

#### 2.1.2 Sexually Risky Behaviors

This section used previous inventory which is related to youth involvement in risky sexual behavior during the past three months. It comprises sexual intercourse, numbers of sex partners, age at first sex experience, and having unprotected sex. This part was already developed by Blum and Mmari (2005) amd Yi et al. (2010). In addition, we asked survey respondents: have you ever experienced pregnancy?

#### 2.1.3 Knowledge and Attitudes towards Sexuality

It was evaluated based on the validated instrument developed by researchers. There are 9 items for measuring adolescent’s knowledge about sexual reproductive health as well as 6 items for measuring their attitude concerning sexual reproductive health.

#### 2.1.4 Psychological Domain

The Asian Adolescent Depression Scale (AADS) (Woo et al.,2004) was used to measure psychological constructs, such as negative self-evaluation and lack of motivation. We also used an adapted scale of self-control from the scale developed by Sutherland et al. (1947) to complete the psychological domain.

#### 2.1.5 Family Domain

This realm comprised family communication and attachment, family positive involvement, and family management practice from the scale developed by Ungemack et al. (2000). Additionally, we measured school attachment using items adapted from a previous study by Yi et al. (2010).

#### 2.1.6 Peer Delinquency

It was evaluated based upon the scale developed by Khaidzir and Hanina (2007). There are 18 items which includes five dimensions namely: peer sexual disorder, drug abuse, discipline, integrity misconduct, and criminal abuse. The constructs and scales were drawn and modified from the previous literature regarding peer delinquency.

#### 2.1.7 Sexual Activity

It was defined as having sexual intercourse outside of marriage in the last three months and respondents were asked, ‘‘Have you ever had sexual intercourse in the last three months?”. However, sexual activity involves behaviors from kissing to sexual intercourse (O’Sullivan & Allgeier, 1998). The term of sexual activity in this study refers to having “sexual intercourse” outside of marriage.

## 3. Results

Of the 770 rural female school students, 3.2% had sexual intercourse (Table 1). The mean age of the respondents was 15. 16 years (SD=1.4; range 13-17) and a majority of them were living with married parents, the father, as head-of-household. Of respondents who report ever having sex, 36% were 17 years old and their parents’ educational level was also relatively low and 68% percent had primary and secondary education. Out of these sexually active adolescents, 68% were living with two parents and their father supported the family as head. It would appear that parental marital status in a rural setting with conservative culture is not protective in terms of reproductive health for adolescents, particularly as far as rural females are concerned. In addition, other factors such as age, socio-economic status, and low educational status of parents may play crucial roles in sexual misconduct matters involving female students in rural areas.

**Table 1:** Socio-demographic characteristics of the rural female adolescents (n=770)

	Number of adolescents	having sexual intercourse	
		Have never had (%) (n=745)	Ever had (%) (n=25)
**Age**		
13	114	15.2	4.0
14	168	21.7	24.0
15	111	14.5	12.0
16	237	31.0	24.0
17	140	17.6	36.0
	770	96.8	3.2
**Parental marital status**		
Married	680	89.1	68.0
Divorced/separated	54	6.3	24.0
Father/mother/both parents died	36	4.6	8.0
**Head of household**		
Father	662	86.6	68.0
Mother	65	8.2	16.0
Grandfather	26	3.2	8.0
Grandmother	3	0.4	-
Elder brother	5	0.7	-
Elder sister	1	0.1	-
Other relatives	8	0.8	8.0
**Head of household’ s education**		
Ever received formal education	20	2.7	-
Primary	80	9.5	36.0
Secondary	359	47.1	32.0
Tertiary	174	22.9	16.0
Religious school	10	1.3	-
Don’t know	127	16.5	16.0

Table 2 shows risky sexual behaviours among the 25 sexually active female adolescent. Of the 25 rural female adolescents, 52% had sex for the first time at the age of 14 years old. Findings indicate that over half of female respondents (52% vs 48%) had forced sex or were raped. Regarding numbers of sex partners, 80% of respondents reported that they had sex with one person and 20% of them had sexual intercourse with more than one. Table 2 presents 72% of respondents who reported ever having sex, had not used contraception during the most recent sexual intercourse. Among the 25 sexually active adolescents, 12% have ever experienced pregnancy. The Chi-square (χ^2^) test proved that there is a significant relationship between marital status of parents and having sexual intercourse among respondents (P<0.01).

**Table 2:** Risky Sexual behaviors among sexually active female adolescents (n=25)

	N	%
Sexual intercourse for the first time	
14	13	52.0
15	3	12.0
16	8	32.0
≥17	1	4.0
Type of first time sex experience	
Voluntary (fun/money)	12	48.00
Involuntary (being forced/raped)	13	52.00
Numbers of sex partners	
One	20	80.0
Two or more	5	20.0
Contraceptive use at last sexual intercourse	
Yes	7	28.0
No	18	72.0
Ever experienced pregnancy	
Yes	3	12.0
No	22	88.0

### 3.1 Results of Bivariate Analyses

Among rural female adolescents, significant factors influencing risky sexual behavior comprised higher level of midnight activities (β=.921, SE=.184, p<0.0001), clubbing (β=.892, SE=.415, p<0.05) and drug abuse (β=-1.788, SE=.828, p<0.05). Similarly, higher level of sexual disorder among peer group members (β=.442, SE=.147, p<0.05), lack of school attachment (β=-.407, SE=.131, p<0.05), incomplete family structure (β=-.403, SE=.155, p<0.01), lower level of family communication (β=.-.382, SE=.125, p<0.05) and negative attitude toward sexual health (β=-.064, SE=.019, p<0.01) affect risky sexual behavior. Table 3 shows that higher level of midnight activities and clubbing was significantly associated with the highest likelihood of hazardous sexual behaviour.

**Table 3:** Results of bivariate regression analyses of risk and protective factors influencing risky sexual behaviour

Expected risk and protective factors	β	SE	*p*- value
Lifestyle-involve in midnight activities	.921	.184	.000
Lifestyle-clubbing	.892	.415	.032
Lifestyle-drug abuse*	-1.788	.828	.031
Peer-sexual disorder	.442	.147	.003
School attachment	-.407	.131	.002
Family structure (0=incomplete; 1=complete)	-.403	.155	.010
Family communication	-.382	.125	.002
Attitude towards sexual health and reproductive	-.064	.019	.001
Lifestyle-tobacco	.248	.260	.340
Lifestyle-alcohol	-.185	.367	.613
Lifestyle-coupling/dating	.125	.116	.282
Lifestyle-illegal motorcycle race	.247	.325	.448
Negative self-evaluation	.137	.103	.185
Self-control	.066	.131	.616
Family management	.052	.030	.087
Knowledge on Sexual health and reproductive	.008	.027	.768
Living arrangement (0=without family; 1=with family)	.009	.138	.948
Family involvement	.070	.124	.577
Peer-discipline	.114	.146	.433
Peer-drug abuse	.001	.169	.995
Peer-criminal	.082	.172	.632
Peer-integrity disorder	.084	.125	.506

Note. SE=Standard error.

* Only 3 respondents have a friend who involved with drugs abuse and all of them are in a group who have ever had sexual intercourse.

### 3.2 Results of Multivariate Logistic Regression

The multiple logistic regression analysis used the full model which deemed all independent variables together. As shown in Table 4, the odds for having premarital sexual intercourse among rural students were controlled by variables namely peer-sexual disorder, attitude towards sexual health and reproductive, midnight activities, and negative self-evaluation. The model explained between 10% (Cox & Snell R Squared) and 41% (Nagelkerke R Squared) of the variance in sexual intercourse.

**Table 4:** Results of multivariate logistic regression for predicting premarital sexual intercourse

Variable	OR	95% CI	
		Lower	Upper
Peer-sexual disorder	5.85**	2.06	16.58
Attitude towards sexual health and reproductive	4.03*	1.35	12.03
Midnight activities	8.75**	3.11	24.62
Negative self evaluation	6.49*	1.45	28.99

* p≤ 0.05

** p≤ 0.001

χ^2^=84.328 (11)**; Cox & Snell R^2^=.104; Nagelkerke R^2^=.416

The odds ratios (OR) for having sexual intercourse among rural female adolescents were significant for peer-sexual disorder (OR= 5.85, 95% CI: 2.06-16.58, P≤ 0.001), attitude towards sexual health (OR= 4.03, 95% CI: 1.35-12.03, P≤ 0.05), midnight activities (OR= 8.75, 95% CI: 3.11-24.62, P≤ 0.001), negative self-evaluation (OR= 6.49, 95% CI: 1.45-28.99, P ≤ 0.05). Although higher level of sexual disorder among peer group members, higher level of midnight activities, and negative self-evaluation and attitude toward sexual health were significant at bivariate analysis, no statistically significant correlation was observed between demographic factors considered in the study and sexual intercourse among rural female teens using multivariate logistic regression.

## 4. Discussion

This research presents the prevalence of the premarital sexual intercourse among rural female adolescents was low (3.2%) compared to sexual intercourse among adolescents from a study conducted by Zulkifli & Low (2000) which was 13% (60 out of 486 and 44 male teens vs. 16 female teen). It was lower than the prevalence of sexual intercourse among local adolescents in previous study done in 1986 which showed 9% (Zulkifli et al.,1995), as well as to the adolescents at Negeri Sembilan, which was 5.4% (Lee et al.,2006). Never the less, all of these percentages are low compared with their western counterparts.

The study displays more than 50% of respondents who admitted ever having sex (13 out of 25), had been raped or forced into sex acts. In Malaysia, females are generally supposed (in media and popular discourse) to be at higher risk of sexual victimization compared with males (Choo et al.,2011). There are protective factors with regard to sexual behavior associated with family domain variables such as family discipline and structure or family communication which can impact on sexual behaviors and victimization. However, the effects of these protective factors on sexual intercourse in rural female teens were null by other factors such as peer delinquency and adolescents’ attitude and self-evaluation along with their midnight activities. It is worth noting that socio-cultural taboos concerning sexual activity in rural areas also limit awareness raising about sexual behaviours in Malaysia.

Bivariate analysis shows that midnight activities and clubbing are the most salient risk factors of sexual behavior among female rural students. Despite the conservative attitude of Malaysian on premarital sexual behavior, major changes have taken place in female adolescents due to the impact of modernization and/or westernization. Consequently, some of the individual risk factors such as smoking, drinking alcoholic beverages, and taking drugs can increase the number of risky sexual behaviours among female teens during midnight activities. This finding is in line with previous Asian reports (Liu et al., 2006; Lee et al., 2006; Wong et al.,2009).

Another explanation could be critical for sexual activity in teens, is their desire for casual sex. It is possible that socio-cultural background and physiological characteristics change sexual desires among older adults (Palacios-Cena et al., 2012).

The results also associate with the effects of potential protective factors such as family structure and family communication. Bivariate linear regression proposes that for rural female adolescents, a better family communication and stable family structure may operate the same as positive attitude towards sexual and reproductive health against risky sexual behaviour. In other words, successful parent discipline and control plays a significant role in the value orientation of the adolescent (Rose, 1999).

Besides, sexual disorder among peer group has significant association with risky sexual behavior among rural girls. This study is in line with previous findings which propose that peer influence appears to be crucial in adolescents’ sexual behaviour (Whitbeck et al., 1992; Brown et al., 1997; Mumari & Blum, 2009; Yi et al.,2010).

In contrast, drug abuse among friends does not play an important role in involving risky sexual behavior. However, substance use is one of the most relevant predictor of risky sexual behavior among teens (Tapert et al., 2001; Yi et al.,2010). Besides, substance use may have a confusing effect on cognitive functions leading to deprived decision-making among adolescents and increases their connection with risky sexual behavior (Bell et al., 2003; Yi et al.,2010). Another possible reason concerns the significance of life style drug abuse in bivariate analyses in this study, is few number of adolescents (n=3) who have friends involving drug.

The results from this countrywide representative sample point out that the higher level of midnight activities negatively affect rural female adolescents and play an important role in predicting sexual intercourse among them. Findings of this study shows that female teens have late-night activities are almost nine times more likely to have sexual intercourse (Table 4).

The odds ratio (OR) for having sexual intercourse in rural girls was not significant for most of protective factors such as family communication and involvement, and family management (Table 4). Despite the family value in Asian culture, it is expected that low parental educational attainment and modernization affect family communication and, simultaneously, the risk factors exaggerate sexual risky behaviours in rural female adolescents in the study.

After controlling for other covariates in the multiple regression model, negative self-evaluation and adolescents’ attitude towards sexual health remained significantly associated with premarital sexual intercourse in rural girls. Results of this study show that sexually active girls are almost seven times more likely to have negative self-evaluation. Likewise, adolescents’ negative attitude regarding sexual health increases the sexual intercourse rate about five times (Table 4). However, the results of the bivariate linear regression shows that adolescents’ attitude towards sexual health was also associated with adolescents’ risky sexual behaviours. Similarly, previous studies showed that troubled adolescents may engage in risky health activities such as substance use to relieve negative psychological states (McKirnan et al.,1996).

These findings suggest training both family members and adolescents in community health programs for youth. These results also recommend researchers continue to search for various risk and protective factors related to risky sexual behaviour and a large community sample of sexually active adolescents.

Peer-sexual disorder is one of the most influential predictors of sexual intercourse among sexually active teenagers in this study. Having experienced sexual disorder among peer group increases six times to have sexual intercourse in female teens in this study (Table 4). Peer delinquency can operate as a source of pressure for adolescents and provide adolescents the opportunity to contribute to poor decision making and becoming involved in the range of sexual behaviour (Keliwer & Murrelle, 2007; Yi et al.,2010).

It is worthy to say that family structure, discipline, and communication are associated with risky sexual behaviors among girls only using bivariate analysis. However, multivariate analysis shows relative risk ratios from adjusted logistic regression and confirms the family domain, as defined here; do not stand a significant predictor of sexual intercourse in rural female adolescents. Although the non-significant association do not prove previous literature which proposes that protective factors are also crucial in adolescent health outcomes (Ostaszewski & Zimmerman, 2006; Yi et al.,2010).

Despite these interesting results, several limitations merit discussion in the current study. First, the number of respondents for logistic regression was small and therefore lacks a robust statistical analysis. Second, the study was a web-based survey and thus technical issues can cause inconsistent responses. Third, our measures related to risky sexual behavior are challenging in the study since sexual activity refers to having sexual intercourse outside of marriage.

Besides, the studied items concerning risky behaviors among sexually active female teens are quite limited. Additionally, the same as all cross- sectional surveys disallow us to investigate causal relationships between the predictors and risky sexual behaviors. Another limitation that should be acknowledged is the representative sample has not met a wide range of social status group and therefore the results may not be generalized to other adolescents with diverse life style in Malaysia.

Finally, the most important limitation that may provide a consideration for future studies is that although this research carried out among a nationally representative sample of rural female adolescents, only 25 students had premarital sexual intercourse. The number may not be accurate which could be argued that shyness is a stereotype among rural Malay girls. Our results propose the importance of considering cultural taboos and tolerance for pre-marital sex among Asian female adolescents when planning and implementing multifaceted intervention and prevention programs.

## 5. Conclusion

The project is designed to deliver more knowledge sharing in relation to risks and protective factors associated with risky sexual behaviors among rural female adolescents in Malaysia. Our findings do mean that the relative risk factors of sexual behaviors (e.g. midnight activities, peer-sexual disorder) are more effective than other protective factors (e.g. family communication, family attachment) among rural female adolescents which may refer the matter to the modernization and transformation. Cost-effective essential health care package in schools may have pivotal role in maintaining youth sexual health behavior and help to prevent teen pregnancy, HIV/AIDS, and other sexual transmitted diseases among adolescents at risk of sexual misconduct. This research supports the voice of rural female students beyond the conservative culture they were raised in which would be the best source of information for awareness-raising for scaling up HIV (Human Immunodeficiency Virus) and STDs (Sexually Transmitted Diseases) prevention in rural districts in Malaysia.
